# Characterisation of Indica Special Protein (ISP), a Marker Protein for the Differentiation of *Oryza sativa* Subspecies *indica* and *japonica*

**DOI:** 10.3390/ijms15057332

**Published:** 2014-04-29

**Authors:** Keming Zhu, Chao Min, Hengchuan Xia, Yanhua Yang, Bin Wang, Keping Chen

**Affiliations:** Institute of Life Sciences, Jiangsu University, Zhenjiang 212013, China; E-Mails: uegzkg@sina.com.cn (K.Z.); xsda2879@gmail.com (C.M.); hchxia@mail.ujs.edu.cn (H.X.); yanhuayang@126.com (Y.Y.); wb-wb628@163.com (B.W.)

**Keywords:** ISP, rice, *indica*, *japonica*, 2-DE

## Abstract

Based on both morphological and physiological traits, Asian cultivated rice (*Oryza sativa* L.) can be classified into two distinct subspecies, *indica* and *japonica*. To better understand the differences between the two subspecies, a proteomic approach was used to profile proteins present in the yellow seedling stage of 10 *indica* and 10 *japonica* rice varieties. We report the discovery of a new protein, Indica Special Protein (ISP), which was only detected in yellow seedlings of *indica* varieties, and was absent from *japonica* varieties. Hence, ISP may represent a key gene for the differentiation of *indica* and *japonica* subspecies.

## 1. Introduction

Rice (*Oryza sativa* L.) is widely cultivated worldwide, providing the caloric needs for more than half of the world’s population, and especially in Asia. As a diploid crop with a relatively small genome, rice provides great potential for increasing our understanding of the genetic mechanisms behind crop domestication and improvement. Completion of the rice genome sequencing has considerably accelerated studies into the history and process of rice domestication [1–4].

Asian cultivated rice can be subdivided into two main subspecies, *indica* and *japonica*, on the basis of various morphological and physiological traits [5–8]. These two subspecies have been recorded as distinct rice groups in Chinese literature since the Han Dynasty (over 2000 years ago) [9]. However, the origins and evolutionary history of the two rice groups is still unclear. Two conflicting hypotheses regarding the origins of the two subspecies have been proposed. The first hypothesis suggests that the *indica* group was originally domesticated from *Oryza. rufipogon* and *japonica* later was derived from *indica* [6,10]; whilst the alternative hypothesis proposes that the *indica*/*japonica* differentiation occurred as a result of their wild ancestors to differing ecological and geographical environments [11–13]. In terms of their ecogeography, *indica* cultivars are widely grown in lowland areas of the tropics and subtropics, whereas *japonica* cultivars are cultivated in both temperate and tropical regions [14,15].

Traditionally, the *indica* and *japonica* cultivars have been distinguished on the basis of their morphological characteristics, including grain shape, apiculus hair length, leaf color or through biochemical assays for reaction to phenol and sensitivity to potassium chlorate [5,6,16]. Despite the numerous reproductive barriers between the *indica* and *japonica* subspecies [6,17], overlaps in the range of variation exist for any one of these phenotypic traits has led to some confusion regarding the classification of particular genotypes [6].

Since publication of the first rice genetic map [18], DNA markers have been widely used to differentiate between *indica* and *japonica* cultivars [7,19]. Isozymes, SSRs (simple sequence repeats), and SNPs (single nucleotide polymorphisms) provide high resolution of the population structure. The availability of complete genomic sequence for both *indica* and *japonica* [1,3,20] has rapidly improved the use of these molecular markers. In addition to archaeological analysis and the use of currently available molecular markers, studies into the key domestication genes in rice will provide novel insights into the dynamics of the rice domestication process [21]. These genes are likely to be associated with grain size, shape, color, fragrance, amylose content and the reproductive barriers between the modern *indica* and *japonica* groups [21,22].

In this study, 10 *japonica* and 10 *indica* varieties were investigated by two-dimensional gel electrophoresis (2-DE). We identified one protein, termed Indica Special Protein (ISP), present in all of the *indica* varieties, but absent from the *japonica* varieties. Hence, our results suggest that ISP is a protein marker for *indica* rice varieties. In addition, the study of ISP protein could also help improve our understanding of the dynamic process of rice speciation.

## 2. Results

### 2.1. Comparative Analysis by Two-Dimensional Gel Electrophoresis (2-DE)

**Figure 1.**
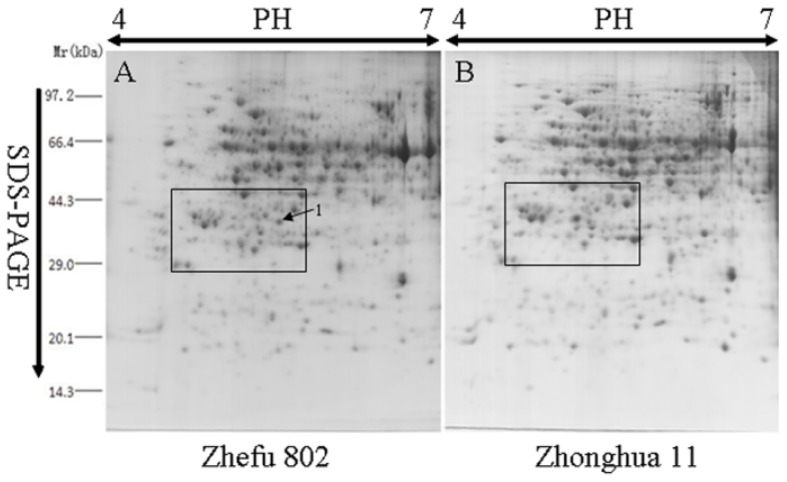
Comparative two-dimensional gel electrophoresis (2-DE) of proteins from Zhefu 802 (*O. sativa* L. ssp. *indica*) and Zhonghua 11 (*O. sativa* L. ssp. *japonica*) yellow seedling. Differentially expressed proteins are indicated by arrows and labeled numerically. (**A**) Zhefu 802; and (**B**) Zhonghua 11. The box in the two maps shows the interception area in Figure 2.

**Figure 2.**
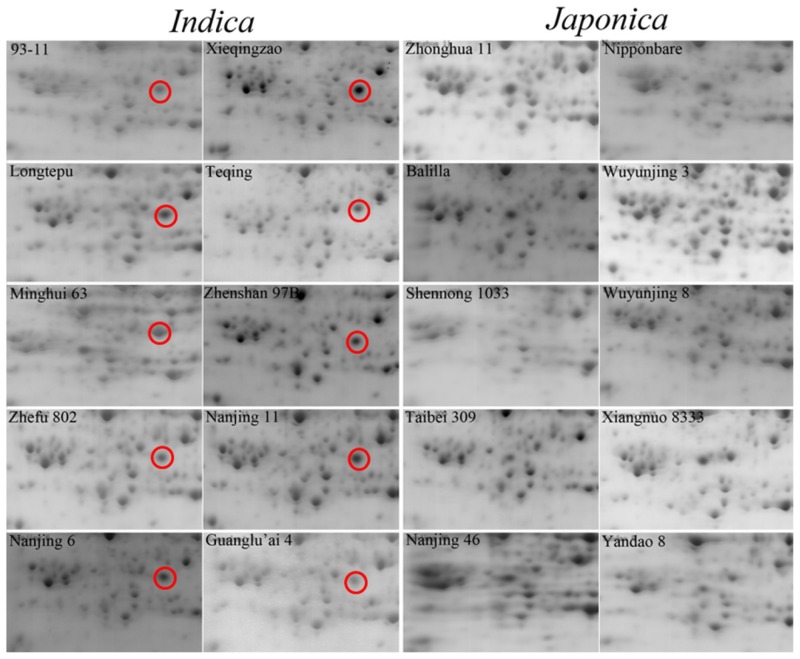
Part of 2-DE of proteins from 10 *indica* and 10 *japonica* varieties yellow seedling. The red circles indicate the unique protein (spot No. 1) detected only in *indica* rice varieties.

There are many high abundance proteins in rice green leaves, including ribulose 1,5-bisphosphate carboxylase/oxygenase (RuBisCO), which accounts for about 50% of total soluble protein contents and affects the detection of some low abundance protein in proteomic analysis [23]. Thereby, we selected the rice yellow seedlings as research materials, which will better contribute to the identification of low abundance proteins and can provide more useful information. To understand the differences between *indica* and *japonica* varieties at the protein level, we employed 2-DE to identify the differentially expressed proteins in 10-day-old dark-treated seedlings of 10 typical *indica* and 10 typical *japonica* varieties (Table S1). Over 700 unique protein spots were identified in the yellow rice seedlings. Most protein spots were located in the 30–95 kDa, pH 4.5–6.5 region. Figure 1 showed the proteomic profiles from Zhefu 802 (*O. sativa* L. ssp. *indica*) and Zhonghua 11 (*O. sativa* L. ssp. *japonica*), which are classical *indica* and *japonica* varieties and are still planted in China. There were no significant differences in the protein expression profiles between *indica* and *japonica*. However, one protein, ISP (spot No. 1), with an approximate molecular weight is about 40.0 kDa and pH of 5, was detected in gels from *indica* varieties, but not in those from *japonica* varieties (Figure 2).

### 2.2. Mass Spectrometry Data Analysis

**Figure 3.**
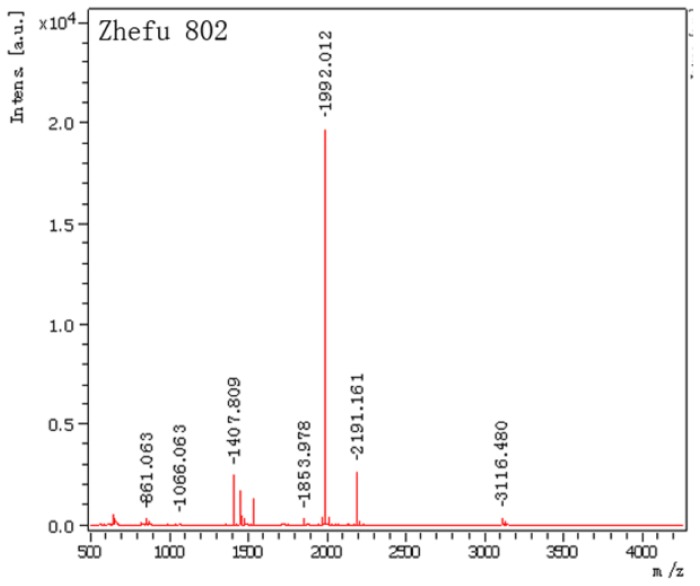
Peptide mass fingerprinting of No. 1 spots extracted from 2-DE gel of Zhefu 802 (*O. sativa* L. ssp. *indica*). Matrix-assisted laser desorption/ionization-time of light mass spectrometry (MALDI-TOF-MS) analysis of tryptic digesting.

The identified ISP spots were excised from 2-DE gels and analyzed by matrix-assisted laser desorption/ionization-time of light mass spectrometry (MALDI-TOF-MS). Figure 3 shows the mass spectrometry data of the identified protein. Details of masses and peptides were listed in Table 1. ISP was identified as a salt-induced protein (salT) with the NCBI accession number gi|115436436, and the gene was located on chromosome 1. The Mascot score was 100 (*p* < 0.05). The molecular weight of salT protein is 15.6 kDa.

### 2.3. Nucleotide Variations and Protein Divergences in Indica Special Protein (ISP) Region

**Figure 4.**
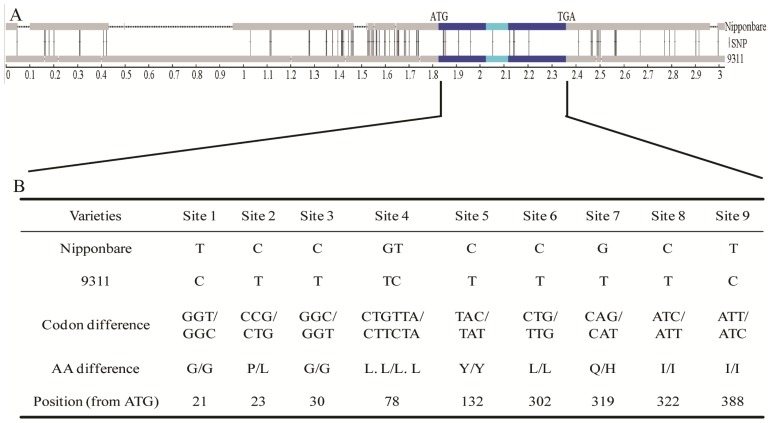
Schematic drawing of *ISP* structure with a summary of DNA and protein polymorphisms in Nipponbare (*O. sativa* L. ssp. *japonica*) and 9311 (*O. sativa* L. ssp. *indica*). (**A**) Gene model of *ISP*. Dark blue boxes indicate the two exons, and blue boxes represent introns and other non-coding regions. Dotted lines show the deletions. Numbering is from the left border of promoter region. SNPs are indicated by solid bars; and (**B**) Summary of the DNA and protein variations in *ISP* between Nipponbare and 93-11.

By searching the KOME cDNA database, we identified a full-length cDNA clone corresponding to the *ISP* gene with size of 438 bp, AK105034 (Rice Genome Research Center of the National Institute of Agrobiological Sciences). Sequence comparison between the genomic DNA and cDNA revealed that the *ISP* gene is composed of two exons and one intron (Figure 4A), and encodes a protein of 146 amino acids. To compare the DNA sequences of *ISP* gene region, we obtained DNA sequences of Nipponbare (*O. sativa* L. ssp. *japonica*) and 93-11 (*O. sativa* L. ssp. *indica*) from NCBI. Seventy-five single nucleotide polymorphisms (SNPs) and 15 insertions/deletions (InDels) were identified within this 3.1kb region (Figure 4A). The majority of the variable sites were found outside the open reading frame in promoter sequences or in the 3′-flanking regions, with only nine SNPs located in coding regions. However, an amino acid change exists at sites 2 and 7 (Figure 4B).

### 2.4. Expression of the ISP Gene

**Figure 5.**
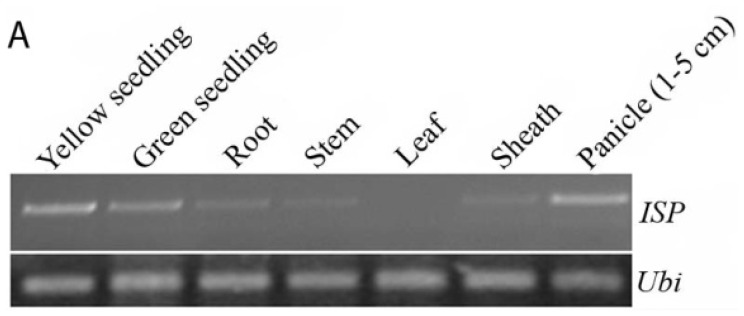

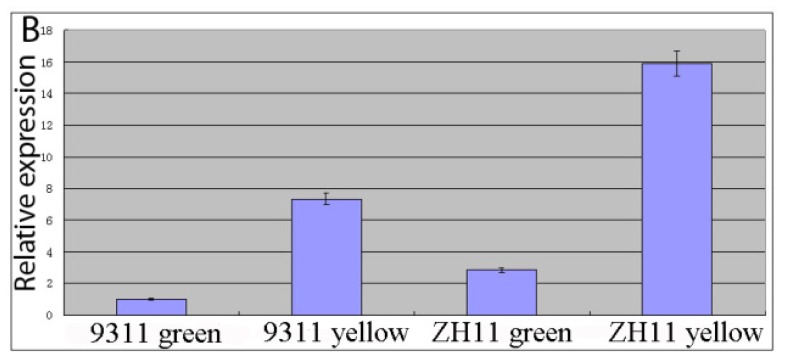
Gene expression pattern analysis of *ISP*. (**A**) Relative expression of *ISP* by real-time polymerase chain reaction (RT-PCR) analysis. Amplification of the rice *Ubiquitin* gene (*Ubi*) was used as a control; and (**B**) Quantitative RT-PCR analysis of *ISP* expression in green and yellow seedling of Zhonghua 11 (ZH11) and 93-11 seedlings.

The ISP protein was detected in gels from the *indica* varieties, but not in those from *japonica* varieties (Figure 2). So, we examined the expression of *ISP* gene in the seedlings of Zhonghua 11 (*O. sativa* L. ssp. *japonica*) and 93-11 (*O. sativa* L. ssp. *indica*). As shown in Figure 5B, more *ISP* mRNAs accumulated in the yellow seedlings than in the green seedlings of both Zhonghua 11 and 93-11, with the greatest abundance detected in the yellow seedling of Zhonghua 11. These results suggest that light may decrease the expression of the *ISP* gene in both Zhonghua 11 and 93-11. However, the No. 1 protein spot could not be detected in yellow seedlings of *japonica* varieties (Figures 1 and 2). The 2-D PAGE (2-dimensional polyacrylamide gel electrophoresis) indicated that the molecular weight of the No. 1 spot protein was approximately 40.0 kDa (Figure 1), higher than the molecular weight of the salT protein. We speculate that in the *indica* varieties, ISP might be the post-translational modification of salT protein.

In order to better understand the function of the *ISP* gene, we examined its expression pattern in various organs, including the roots, stems, leaves, sheath, and panicles of Zhonghua 11 at the heading stage, and in seedlings. We performed semi-quantitative real time polymerase chain reaction (RT-PCR) analysis to estimate *ISP* transcript level. As shown in Figure 5A, *ISP* is mainly expressed in the seedlings and panicles, especially in the yellow seedlings. We could also detect the expression of *ISP* in the roots, stems and sheath, but not in the leaves. It has similar expression pattern in 93-11 (Figure S1). These results showed that the *ISP* gene differs in its spatial and temporal expression.

## 3. Discussion

Asian rice (*Oryza sativa* L.) has two genetically divergent cultivars, *indica* and *japonica*, and ecologically distinct wild progenitors, *O. nivara* and *O. rufipogon* [24,25]. The genetic divergence between the *indica* and *japonica* groupings may represent independent domestications from divergent pools of *O. rufipogon* which have differentiated over thousands of years of geographical isolation [26].

In this study, we carried out proteomic analysis to globally identify proteins related to the *indica* and *japonica* subspecies. Using 10 *indica* and 10 *japonica* varieties 2-D PAGE profiles, we detected a protein, ISP, present in the yellow seedlings of *indica* varieties but not in *japonica* varieties (Figure 2). The ISP protein was identified as a salt-induced protein (salT) in rice. The salT protein has previously been isolated and characterized from the roots of rice (Taichung native 1) treated with salt [27]. In general, the salt tolerance of *indica* varieties is reported to be greater than that of *japonica* varieties [28,29]. The presence of the ISP protein in *indica* varieties alone may thus explain its higher salt tolerance.

We observed that the molecular weight of ISP was around 40.0 kDa in our 2-DE gels (Figure 1), higher the molecular weight of salT protein (15.6 kDa). Hence, it is possible that in *indica* varieties the ISP protein might be the post-translational modification of salT protein. As shown in Figure 4B, two amino acids (P8–L8, Q74–H74) differ between *indica* and *japonica*. We speculate that these two amino acids were important for salT protein post-translational modification.

Londo *et al.* examined the geographical distribution of the sequence haplotypes at three genetic loci using a large collection of wild and domesticated rice, and showed that the rice subspecies separation was enforced by significant geographical barriers in addition to the genetic sterility barriers [30]. It is suggested that environmental factors have played an important role in the domestication of *indica* and *japonica* varieties. In our study, we found that the *salT* mRNA accumulated more in the yellow seedlings than in the green seedlings (Figure 5B). Previous studies have also shown that the *salT* expression increased rapidly after wounding and salt, dehydration or ABA treatment in rice [31–34]. Thus, we suggest that the *salT* gene may respond to environmental factors. Previous studies also showed that high levels of amino acid variation found in genes rapidly diverged between species. For example, four hybrid incompatibility genes in *Drosophila* (*OdsH*, *Hmr*, *Nup96* and *Lhr*) showed high levels of amino acid variation and have been attributed to positive selection [35–38]. Concerted evolution and positive selection have also rapidly altered the sequence of a hybrid sterility gene *Prdm9* in mice [39], the reproductive barrier gene *S5* in rice [22]. In the *ISP* gene, 75 SNPs and 15 InDels were identified, and two amino acids were changed (Figure 4). These results suggest that the *ISP* gene may play important role in the domestication of *indica* and *japonica* rice varieties. However, the detailed molecular mechanism still needs to be explored.

Thus, tISP is not only involved in response to environmental factors, but also plays an important role in the differentiation of *indica* and *japonica* rice varieties. Complete understanding of the function of this protein requires further detailed characterization.

## 4. Experimental Section

### 4.1. Plant Material

Rice varieties used in this experiment were showed in Table S1. After germination, all the rice seeds were cultured at 28 °C in the dark. Then, the seedlings were collected at the tenth day. Samples were frozen in liquid nitrogen immediately, and stored at −80 °C.

### 4.2. Protein Sample Preparation

The yellow seedling was grounded in liquid nitrogen, and was extracted using buffer containing 20 mM Tris–HCl, pH 7.5, 250 mM sucrose, 10 mM EDTA, 1 mM phenylmethylsulfonyl fluoride (PMSF), 1 mM β-mercaptoethanol and 1% (*v*/*v*) Triton X-100, as described by Cilia *et al.* [40]. Briefly, the mixture was eddied for 30 min and centrifuged. The supernatant was collected and Tris-saturated phenol was added to precipitate proteins. The phenol layer containing proteins was collected, incubated with methanol solution (containing ammonium acetate) and centrifuged to pellet proteins. The pellet was washed with methanol acetone (containing 0.7% dithiothreitol (DTT)), lyophilized, and dissolved in solution containing 7 M urea, 2 M thiourea, 4% (*w*/*v*) chaps and 1% (*w*/*v*) DTT and centrifuged. The supernatant, as the sample of total yellow seedling protein, was pooled and stored at −80 °C for later use. The protein concentration was determined using RC DC™ (Bio-Rad, Hercules, CA, USA) kit.

### 4.3. Two-Dimensional Electrophoresis (2-DE)

Two-DE was performed with 17 cm (linear, pH 4–7) immobilized pH gradient (IPG) gel strip (Bio-Rad), according to Kim *et al.* [41]. A total of 1200 μg yellow seedling protein was loaded onto IPG strip using active rehydration (13 h with 50 V), and the isoelectric focusing (IEF) was performed at 17 °C with a voltage gradient of 250 V for 0.5 h, 1000 V for 1 h, 10,000 V for 5 h, then continued for a total of 60,000 Vh. The focused strip was equilibrated for 15 min with equilibration solution (6 M urea, 0.375 M Tris-HCl, 20% (*v*/*v*) glycerol, 2% (*w*/*v*) sodium dodecyl sulfate (SDS)) containing 2% (*w*/*v*) DTT, then was equilibrated for another 15 min with equilibration solution containing 2.5% (*w*/*v*) iodoacetamide. Equilibrated strip was then sealed on the top of 12% SDS-PAGE gel for electrophoresis. The gel was visualized with 0.1% coomassie brilliant blue (CBB) R-250, and scanned with a high precision scanner (ScanMaker 9700XL, Microtek, Shanghai, China) at a resolution of 600 dpi. Spot analysis was performed using PDQuest (version 8.0.1, Bio-Rad).

### 4.4. In-Gel Digestion and Mass Spectrometry Analysis

The in-gel digestion and mass spectrometry (MS) were performed as described by Liang *et al.* [42]. Protein spots were excised from gel, washed with water, distained by sonication in 25 mM ammonium bicarbonate and 25% acetonitrile, dehydrated with acetonitrile, and dried in vacuum. The dried proteins spots were treated by 10 mM DTT for 1 h at 56 °C, alkylated with 40 mM iodoacetamide for 45 min at room temperature, washed with 25 mM ammonium bicarbonate, dehydrated with acetonitrile, and incubated with 3 μL trypsin solution (20 μg/mL) at 37 °C for overnight to completely digest proteins.

The digested proteins were collected and mixed with 10 mg/mL matrix (α-cyano-4-hydroxycinnamic acid, Sigma) dissolved in 50% acetonitrile containing 0.1% trifluoroacetic acid. The mixture was analyzed with matrix-assisted laser desorption/ionization-time of light mass spectrometry (MALDI-TOF-MS) (Bruker, Karlsruhe, Germany). Standard peptide from manufacturer was used as external standard for calibration, and the peptide ions generated by autolysis of trypsin were used as internal standards.

### 4.5. Protein Identification

Mass spectrometry (MS) data were analyzed using MASCOT (Matrix Science, London, UK) and NCBI eukaryotic protein sequence database. The parameter was set as follows: missed cleavages was one, fixed modification was acetylation of carbamidomethyl (C), variable modification was oxidation of methionine (M), mass tolerance was 0.3 Da, mass value was MiH^+^. As described by Zhou *et al.* [43], the protein with a minimum ion score of 79 (*p* < 0.05) was considered to be reliably identified.

### 4.6. Gene Expression Analysis

Total RNA was extracted from various tissues (roots, internodes, leaf, sheath, and panicles) of Zhonghua 11 at the heading stage, and the green and yellow seedling of Zhonghua 11 and 93-11, using a TRIpure reagent (BioTeke, Beijing, China) as described by the supplier. For semiquantitative RT-PCR analysis, total RNA (3.5 mg) was treated with Rnase free DNase, and first-strand cDNA was synthesized through reverse transcription by an oligo (dT) primer (TaKaRa, Dalian, China). Subsequently, the first-strand cDNA was used for PCR amplifications with the following gene-specific primer pairs: 5′-ATGACGCTGGTGAAGATTGG-3′ and 5′-ATGGGTTCCAGAAATCTCCTT-3′ for *ISP*, and 5′-CAAGATGATCTGCCGCAAATGC-3′ and 5′-TTTAACCAGTCCATGAACCCG-3′ for *Ubi*. The PCR samples were collected after 30 cycles for *ISP* and 25 cycles for *Ubi.*

The qRT-PCR was performed on a cycle apparatus (Bio-Rad) using the SYBR Green PCR Master Mix (Tiangen, Beijing, China) according to the manufacturer’s instructions. Amplification was conducted in 96-well optical reaction plates with the following protocol: 94 °C for 4 min, 40 cycles of 94 °C for 15 s, 55 °C for 15 s, and 72 °C for 15 s. Expression levels of target genes were quantified using the Bio-Rad CFX96 real-time PCR detection system (Bio-Rad) by a relative quantization method (DD cycle threshold). The statistical significance was analyzed by Student’s *t* test. Data were presented as mean values of at least two biological repeats with SE.

## 5. Conclusions

In this paper, we identified a protein, ISP, detected only in *indica* varieties and not in *japonica* varieties. In addition, we reported that light may decrease the expression of *ISP*, and that many SNPs and InDels had been found in the *ISP* gene region. Hence, ISP could be used as a marker protein for the differentiation of *indica* and *japonica* rice varieties and may play an important role in the domestication of rice. The protein marker is being used in ongoing research and breeding.

## Supplementary Information



## Figures and Tables

**Table 1.** Protein identified by MALDI-TOF-MS from Zhefu 802 (*O. sativa* L. ssp. *indica*).

Spot No.	Protein name	Monoisotopic masses	Matched peptides
1	salt-induced protein (salT) (score: 100 protein sequence coverage: 66%)	1535.9189	K.KLLGVTIYSSDAIR.S
		1407.8090	K.LLGVTIYSSDAIR.S
		3116.4797	R.SIAFNYIGVDGQEYAIGPWGGGEGTSTEIK.L
		2191.1609	K.EISGTHGPVYDLADIVTYLK.I
		1992.0117	K.EFSIPLQDSGHVVGFFGR.S
		1455.8126	R.SGTLIDAIGIYVHP.
