# Supplementary material for: Characterisation of Indica Special Protein (ISP), a Marker Protein for the Differentiation of Oryza sativa Subspecies indica and japonica
Source: Int J Mol Sci. 2014 Apr 29;15(5):7332–43. doi: 10.3390/ijms15057332 (PMC4057675; doi:10.3390/ijms15057332)
Supplement: Supplementary file 1 [file ijms-15-07332-s001.pdf]

Supplementary Information

Table S1. The rice varieties used in this study.

No.	Varieties	Subspecies	No.	Varieties	Subspecies
1	Zhefu 802	*indica*	11	Zhonghua 11	*japonica*
2	93-11	*indica*	12	Xiangnuo 8333	*japonica*
3	Nanjing 6	*indica*	13	Nipponbare	*japonica*
4	Nanjing 11	*indica*	14	Nanjing 46	*japonica*
5	Guichao 2	*indica*	15	Shennong 1033	*japonica*
6	Xieqingzao	*indica*	16	Wuyunjing 8	*japonica*
7	Zhenshan 97B	*indica*	17	Wuyujing 3	*japonica*
8	Longtepu	*indica*	18	Balilla	*japonica*
9	Teqing	*indica*	19	Taibei 309	*japonica*
10	Minghui63	*indica*	20	Yandao 8	*japonica*

Figure S1. Relative expression of *ISP* by RT-PCR analysis. Amplification of the rice *Ubiquitin* gene (*Ubi*) was used as a control. Total RNA was extracted from various tissues (roots, stems, leaves, sheath, and panicles) of 93-11 at the heading stage, and seedlings of 93-11.

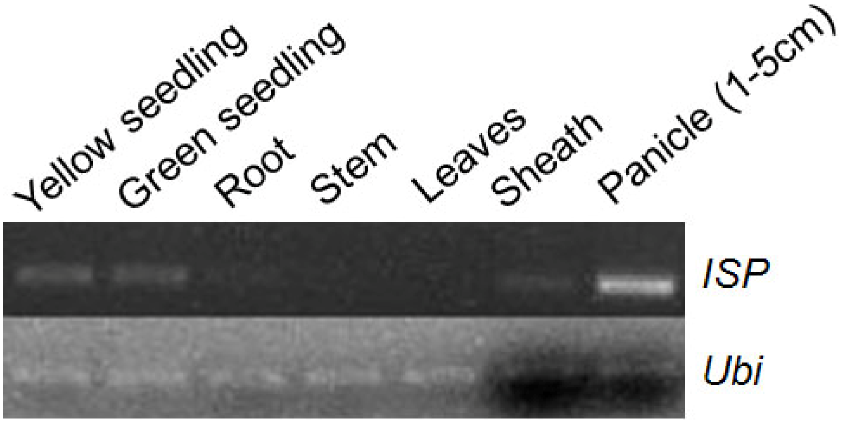

The 3.1 kb sequence of *ISP* gene region from 93-11. The red upper letters show the exon

caggtgctgctcaaaacaacggttaagcagatatccgtcaattagatagtgttacatccgtctttaggctggtgttataattagtttgcttgctatccatcaagatctgtaacgcgt
ggtctttcgagctgaaaactatggactctttgaaacaaagaaaatattaaatcctattattcaaagatatataaatatttagaaagatattaatgttattaaactttgacttacttaaaa
caagtccaaaactgcatgtccctaaatcgccagaagataaggaacacctgtacccgtgataacagaggggtatgaatttgaacacgaggcttctttggcagacgtggcgct
gagtgagcttggctcgcttggtcaaactccgtgcagggacattcagttagctagcagcattatcgacaataaggtagcctttaaggcccatttgaatcgcaggaatgagaaa
acatagaagtaggaaaaacgcaggattttaataggaatgtaagtgtaaaacagagaattgcaaaacacagaaaaaatagaggaatggtcgtttgattggactgcaggaaaa
acacaggaattagaggagagataaagactcaaaggaaagtttccaagaggttttatctcatgttagaattcctccaaaacttacatggcattagccaatctataggaatttcata
ggattccataagatccattcctttgattcaaatggctatataggaaaaaatcctataggaataaaatcctctaaaattcctatgaatttcctttgaatcaaaggtggcctaaatgtta
cttactccgtttcacaatataagactttctagcatttttcatattcatttagatgttaatgaatctaaacatatgtatatgtttagattcattaacatctatatgtatatggataatgctaga
aagtcttacatcgtgaaatggatggagtagcactcaccagcttgtcaaaaaccaaggcttggtgacggcggcttcagaatgaaggatagatggataaatgtctagaatattat
aaagtccaacaaaagatggagcccatgcatgaaagattacgtacacgaatgcagttgatacagtggatgttaggcataagaagcactataaatagagggtgcaatccccat
tgccctacacaactacacaagccgactatcatcacaaggaaatttaagcgaccacgaaggtatgaaagcatagcagtactctgcatttttttttgatgttgttctagctagctct
gcttaaggttttcctttctttcgttctttgtttttttgggtaagctctgctagttgcatgcaatttagattttatccttttacagttggaaaaacatcccaacaaatattaccatgcatgcat
agagattttaggaagctacacatcgggtgaccgattccttaaaaaaaaatctgatggtcaaatcgttgttattttatctataaatttatcaaaactaaaaattttgattaagaaaaac
ataaacggtttataatatgaaatgaagggagtatatgtttgctttcatgaaagttacttgtgcacattaattaaaaaccatagaaatgattaggttacccctacatatttcggaaaca

ataccatatttgttcatgagggtgcaagcattcaaattgatttatatctgaacaaaactgagtaggaataccgtgagcaaacttgacgtttccaaaataatttatatttatgcaaaa
attcacaacttcaaacaagctaacctgaaaaatcatgtttgaatttactaagatgtgcttttgtatttactaaacagagtATGACGCTGGTGAAGATTGGCCT
GTGGGGTGGAAATGGAGGGTCAGCTCAGGACATCAGTGTGCCACCCAAGAAGCTTCTAGGCGTGACAAT
CTACAGCTCAGATGCAATCAGATCCATTGCCTTCAACTATATCGGTGTGGATGGACAGGAATATGCCATTG
GTCCATGGGGTGGGGGCGAAGGCACCTCTACAGaggtaatttacactgcttaattaaggttaagcacttaatttacaaggtgattcctataatag
cagaaataatccaacaaattaaacgatgttactgcAGATTAAATTGGGCTCCTCTGAGCATATTAAGGAGATTTCTGGAACCCA
TGGCCCAGTCTATGATCTGGCTGACATTGTCACCTATCTTAAGATCGTGACAAGTGCTAATAATACATACG
AGGCTGGAGTCCCAAATGGAAAGGAATTCAGCATTCCACTGCAAGACTCTGGCCATGTCGTTGGATTCTT
TGGAAGGTCTGGAACGCTTATCGACGCAATTGGCATCTACGTCCACCCTTGAttcccagtggtcaaagaattactacctact
accatatctacgaaataatgtgccatggtgttgttgtacttcatgcaatccccccattgtctgtgtacgtgttgtaccggtccgtagtacaataaagttggtgatgtatgttcccca
gttgactctttaatatttactacaataagttggggtcatttgtacatgttcagcacctggtccgctcactttatgtgtttctcatattggtatcactatccatgggcaactaatcccttct
tctataacgttattacctactaattacaaataagatcctaaagcataaacctcaatgttccatgtcatcatcagttctcaaccattggatcagtgaattagaccgcttctcagccgtc
ggatgaagaaaatgcagcacactccgattgaaggtagcataaccattatctcacacataggaagcacctgaggcccaggtaatagcttggaccacgtgaggcccatctaa
ttcgattcaggcccaatcaatagtgaaggattcatggtttgtttttggattggtcgagtgtcctactggtgttgactggtgactatgactgcttttgtccatcgacggcaggcggg
actgtagtgcacgccgcacatgcgcgggcaggactgtagtgcacgccgcgcatggtg

The *ISP* gene region sequence from Nipponbare. The red upper letters show the exon

caggtgctgctcaaaacaacggttaagcagatatccgtcaatttgatagtaagatctgtaacgcgtggtctttcgagctgaaaactatggactctttgaaacaaagataatatta
tattaaattctattattcaaagatatctaaatatttagaaagatattaataatgttattaaactttgacttacttaaaacaagtccaaaactgcatgtccctaaatcgccagaagataa
ggaacacctgtacccgtgataacagaggggtatgaaatttggacacgaggcttctttggcagacgtggcgctgagtgagcttggctcgcttggtcaaactccgtgcaggga
cattcagttagctagctagcagcattgtcgacaataagatagcctttaaatgttagcactcaccagcttgtcaaaaaccaaggcttggtgacggcggcttcagaatgaaggat
agatggataaatgtctagaatattataaagtccaacaaaagatggagcacatgcatgaaagattacgtacacgaatgcagttgatacagtggatgttaggcataagaagcac
tataaatagagggtgcaatccccattgccctacacaactacacaagtcgactatcattacaaggaaatttaagcgaccacgaaggtatgaaagcatagcagtactctgcattt
ttttttttgatgttgttctagctagctctgcttaaggttttcctttctttcgttctttgttttttttttgtaagctcaactagttgcatgcaatttagattttatccttttacagttggaaaaacat
ccctataaatattaccatgaatgcatagagattcgaggaagctacaaattggacgactgattccaaaaaaaaaaaaaaaaatcagatggtcacatcattgctattgttttgtgaaa
gtacaaaagcactcgttcggattcaaattacttgtgcaaattaattaaaaaccatagaaatgatcatgttacccctacacatttcggaaacaataccatatatgttagtgtgcgat
cattcaaattgatttatatctgaacaaaactgagtgggaatacggtgagcaaacttgacgattccaaaataatttatatttaggcaaaattttacaacttcaaagttcaaacaagct
aacctgaaaaatcatgtttgaatttactaagatgtgcttttgtatttactaaacagagtATGACGCTGGTGAAGATTGGTCCGTGGGGCGGAAA
TGGAGGGTCAGCTCAGGACATCAGTGTGCCACCCAAGAAGCTGTTAGGCGTGACAATCTACAGCTCAGA
TGCAATCAGATCCATTGCCTTCAACTACATCGGTGTGGATGGACAGGAATATGCCATTGGTCCATGGGGTG
GGGGCGAAGGCACCTCTACAGaggtaatttacactgcttaattaaggttaaccacttaatttacaaggtgattcctataatagcagaaataatccaacaaatt
aaacgatgttactgcAGATTAAACTGGGCTCCTCTGAGCAGATCAAGGAGATTTCTGGAACCCATGGCCCAGTCTA
TGATCTGGCTGACATTGTCACCTATCTTAAGATTGTGACAAGTGCTAATAATACATACGAGGCTGGAGTCC
CAAATGGAAAGGAATTCAGCATTCCACTGCAAGACTCTGGCCATGTCGTTGGATTCTTTGGAAGGTCTGG
AACGCTTATCGACGCAATTGGCATCTACGTCCACCCTTGAttcccagtggtcaaagaattactacctactaccatatctacgaaataatgt
tccatggtgttgttgtacttcatgcaatccccccattgtctgtgtacgtgttgcaccgatccgtagtacaataaagttggcgatatatatgttgtcccagtcgactttttaatatttatt
actacaataagttggggtcatttgtacatgttcagcacctggtccgctcccttgtgtgtttctcatattggtatcactatccatgggcaactaatcccttcttctataacgttattacct
actaattacaaataagatcctaaagcgtaaacctcaatgttccatgtcatcatcagttctcaaccattggatcagtgaattagaccgcttctcagccgtcggatgaagaaaatgc
agcacactccgattgaaggtagctcaaccattatctcacacataggaagtacctgaggcccaggtaatagcttggaccacatgaggcccatctaattcgattcaggcccaat
caatagtgaaggattcatggtttgtttttggattggtcgagtgtcctaccggtgttgagtggtgactatgactgcttttgtccatcgacggcgggcgggactgtagtgcacgcc
gcgcatggtg

© 2014 by the authors; licensee MDPI, Basel, Switzerland. This article is an open access article distributed under the terms and conditions of the Creative Commons Attribution license (http://creativecommons.org/licenses/by/3.0/).
